# Association of Maternal and Cord Blood Choline and Betaine Concentrations with Birthweight: A Prospective Mother–Infant Cohort Study

**DOI:** 10.3390/nu18091456

**Published:** 2026-05-01

**Authors:** Sumiya Aihemaitijiang, Jiaxing Wen, Kai Li, Haoran Ren, Hongtian Li, Yubo Zhou, Jianmeng Liu

**Affiliations:** 1 Institute of Reproductive and Child Health/National Health Commission Key Laboratory of Reproductive Health, Peking University Health Science Center, 38 Xueyuan Rd., Beijing 100083, China; 1410606101@pku.edu.cn (S.A.); jiaxinstudy@163.com (J.W.); 2010306105@stu.pku.edu.cn (K.L.); 2311110183@pku.edu.cn (H.R.); lihongtian@pku.edu.cn (H.L.); liujm@pku.edu.cn (J.L.); 2 Department of Epidemiology and Biostatistics, School of Public Health, Peking University Health Science Center, 38 Xueyuan Rd., Beijing 100083, China; 3 Center for Intelligent Public Health, Institute for Artificial Intelligence, Peking University, Beijing 100871, China

**Keywords:** pregnancy, choline, betaine, infants, birthweight

## Abstract

**Background:** Experimental studies indicated that maternal choline and betaine status have the potential to alter fetal growth, but epidemiological data remain sparse. **Objective:** We aimed to investigate the association of maternal and cord blood choline and betaine concentrations with birthweight outcomes. **Methods:** This prospective cohort study involved 988 mother–infant dyads from Hebei and Shandong provinces. Plasma concentrations of choline and betaine in maternal late pregnancy and cord blood were quantified using ultra-performance liquid chromatography–mass spectrometry. Multivariable linear or logistic regression was performed to examine their association with continuous or binary birthweight outcomes. **Results:** Maternal plasma choline and betaine concentrations in late pregnancy (median [interquartile range]; 12.34 [10.13, 14.78] and 14.99 [12.01, 18.36] μmol/L) were significantly lower than those in cord blood (29.98 [24.74, 35.93] and 31.14 [25.56, 37.28] μmol/L). Each 1 μmol/L increase of late-pregnancy and cord blood betaine concentrations were associated with 9.87 g (95% confidence interval [CI]: −16.08, −3.66 g) and 5.29 g (95% CI: −8.52, −2.06 g) lower birthweight, respectively. Compared with the lowest quintile, the highest quintiles of late-pregnancy and cord blood betaine concentrations were associated with lower risks of large-for-gestational-age (adjusted odds ratios [ORs] = 0.47 [95% CI: 0.24, 0.90] and 0.31 [95% CI: 0.17, 0.56]) and macrosomia (adjusted ORs = 0.12 [95% CI: 0.03, 0.43] and 0.15 [95% CI: 0.05, 0.40]). These associations, particularly for cord blood, persisted and appeared more pronounced in pregnancies with maternal overweight/obesity or gestational diabetes mellitus (GDM), but the interaction effect did not reach statistical significance. No significant associations were observed for choline in any periods. **Conclusions:** Higher plasma concentrations of betaine in maternal late-pregnancy and cord blood were associated with lower birthweight. These findings emphasize the importance of sufficient betaine status during pregnancy, especially among mothers with obesity or GDM.

## 1. Introduction

Birthweight, a key indicator of intrauterine growth, plays a critical role in both short- and long-term health outcomes [1]. Both low birthweight (LBW; <2500 g) and macrosomia (>4000 g) are linked to adverse health outcomes, including neonatal mortality, childhood malnutrition, and chronic metabolic cardiovascular diseases in adulthood [2,3]. Identifying the intrauterine determinants of birthweight is crucial for alleviating the global burden of LBW and macrosomia, especially as progress in reducing their prevalence has been inadequate over the past two decades [4,5].

Maternal choline and betaine status have received growing attention for their potential role in modulating birthweight. Choline is an essential nutrient critical for cell membrane biosynthesis, epigenetic modifications, and neurotransmission [6,7]. Although choline can be synthesized de novo in the body [8], adequate dietary intake remains necessary throughout one’s lifespan, particularly during pregnancy/lactation, when demand increases to support rapid fetal and infant growth [9]. Betaine (trimethylglycine) can be endogenously synthesized via the oxidation of choline [10], a reaction requiring riboflavin and niacin as cofactors, and acquired exogenously through dietary intake. It functions primarily as an osmolyte and methyl donor [11].

Accumulating evidence from animal studies indicates a potential role of maternal choline and betaine intake in regulating fetal growth [12,13,14,15]. Specifically, these nutrients may regulate fetal fat accumulation through modulating placental glycogen and lipid storage [16,17], the expression of placental macronutrient transporters [13,15,18], and fetal obesity-related gene phenotypes [19,20]. Notably, prenatal choline and/or betaine supplementation exerts a bidirectional effect on birthweight in animal models: it prevents fetal overgrowth in obese mice [13,14], while supplementation during early and/or late gestation increases birthweight in Chios ewes [21]. Despite the experimental evidence, epidemiologic data on the association of maternal choline and betaine status with birthweight remain limited.

A few human studies investigated the relationship but yielded conflicting findings [22,23,24,25,26]. For instance, one study reported inverse associations between maternal betaine status and birthweight [22], one found no association [25], and another reported a positive association [24]. Three of these studies focused exclusively on maternal status in mid-pregnancy [22,23,24], and two studies on that in both late-pregnancy and cord blood [25,26]. There is a paucity of studies examining the associations of choline and betaine concentrations both throughout the entire pregnancy and in umbilical cord blood with birthweight. In addition, although animal experiments have indicated a potential bidirectional regulatory effect of maternal choline and betaine status on birthweight, the potential modifying role of maternal body mass index (BMI) has scarcely been investigated.

Therefore, using data from two prospective mother–infant cohorts, we examined the associations of maternal plasma and umbilical cord blood choline and betaine status with birthweight. We additionally investigated the modifying role of maternal BMI and gestational diabetes mellitus (GDM) status in the associations.

## 2. Materials and Methods

### 2.1. Study Design and Participants

This prospective study used data from two mother–infant cohorts. Cohort I was derived from a randomized controlled trial (RCT) evaluating the health effects of micronutrient supplementation during pregnancy. In the RCT, 18,775 singleton nulliparous women with a gestational age of <20 weeks were recruited from five counties in Hebei province during 2006–2009. They were randomly assigned to receive daily supplementation of folic acid, iron–folic acid, or multiple micronutrients until delivery. During the RCT implementation, 690 women from Laoting and Yuanshi counties agreed to collect blood samples. Maternal venous blood was collected during late pregnancy (29.3 [26.23, 32.10] weeks of gestation), and umbilical cord blood was collected during delivery. The maternal and infant characteristics of the included mother–infant dyads (*n* = 690) were generally comparable to those of the original RCT cohort (Supplementary Table S1). The RCT was approved by the Institutional Review Board of Peking University Health Science Center (NO.00001052-09075).

Cohort II recruited 324 singleton pregnant women in late pregnancy from Huantai Maternal and Child Health Hospital, Shandong Province, China, in 2023. Inclusion criteria for participants were ① aged 18–40 years; ② singleton pregnancy; ③ gestational age ≥ 37 weeks; ④ planned to give birth at that hospital; and ⑤ provided informed consent. Exclusion criteria were ① prenatal diagnosis of fetal abnormalities, such as fetal distress and malformations and ② abnormal umbilical cord hindering cord blood collection or the mother planned to retain umbilical cord blood. Following enrollment, all participants were required to complete a structured questionnaire on sociodemographic information, and venous blood samples were collected at enrollment (38.98 [38.48, 39.47] weeks of gestation), as well as umbilical cord blood samples at delivery. This study was approved by the Institutional Review Board of Huantai Maternal and Child Health Hospital (NO.2023-001). Written informed consent was obtained from all participants.

**Figure 1:**
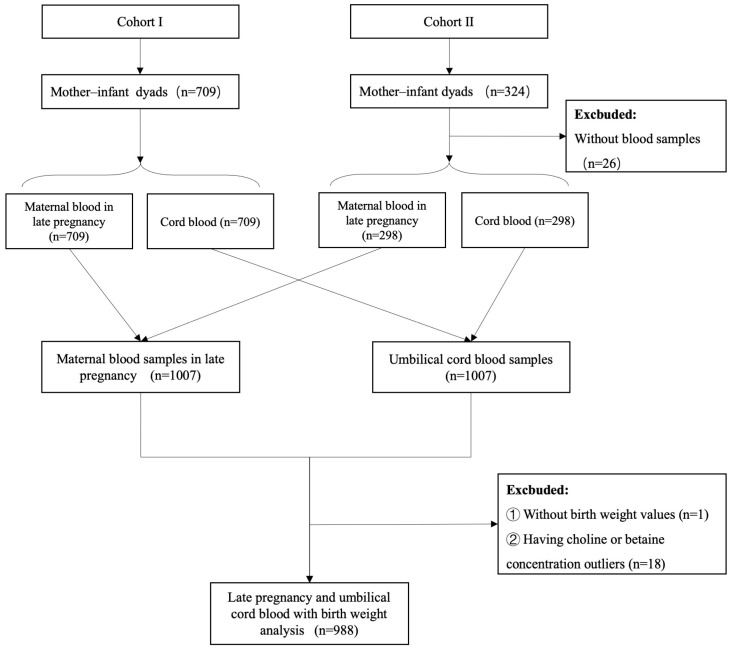
Flowchart of participants included for analysis from Cohorts I and II.

In the present study, we excluded mother–infant dyads with missing blood samples (*n* = 26), missing birthweight data (*n* = 1), or outliers in terms of choline or betaine concentrations (*n* = 18; outliers were defined as values exceeding the mean ± 3 standard deviations [SD]). Finally, an analytical dataset was constructed, which involved 988 dyads from Cohorts I and II to examine the associations of maternal late-pregnancy and umbilical cord blood choline and betaine concentrations with birthweight (Figure 1).

### 2.2. Choline and Betaine Concentration Assessments

Venous blood was collected in EDTA anticoagulant tubes, immediately placed on ice, and centrifuged at 4 °C, 3000× *g* for 10 min within 30 min of collection. Plasma was separated and stored at −80 °C at the national health commission key laboratory of re-productive health at Peking University until analysis in 2025. Plasma concentrations of choline and betaine were quantified using ultra-performance liquid chromatography–mass spectrometry (UPLC/MS–MS) (Waters TQ-S micro, Waters Corp., Milford, MA, USA), with modifications based on the methods described by Holm et al. [27] and Xiong et al. [28]. Briefly, 30 μL of plasma was mixed with 90 μL of deproteinizing solution (acetonitrile) containing the internal standard (Cho-2H9; CP: 95%; 98% atom; and zzstandard^®^). The mixture was vortexed for 1 min to ensure thorough mixing and centrifuged at 15,000× *g* for 20 min at 4 °C. An 80 μL aliquot of the supernatant was filtered through a 0.45-μm filter membrane and transferred to a sealed injection vial for analysis. The analysis was performed on a Shim-pack GlST Amide column (100 mm × 2.1 mm, 3 μm) at 25 °C, with a mobile phase comprising Phase A (acetonitrile) and Phase B (15 mmol/L ammonium formate, pH 3.5). The flow rate was 0.2 mL/min and the injection volume was 2 μL. Elution was carried out using a linear gradient program as follows: 0–0.1 min, 92% A/8% B; 5 min, 70% A/30% B; 5.5–7.5 min, 5% A/95% B; and 12 min, 92% A/8% B. Decluttering potential and collision energy were optimized for each biomarker and its corresponding internal standard. Duplicate quality control samples were included in each analytical batch to assess laboratory accuracy. The intra-day coefficients of variation were 2.4% for choline and 6.7% for betaine, respectively.

### 2.3. Outcomes and Covariates

Birthweight for Cohort I was extracted from the RCT database, which was obtained from hospital delivery records, while birthweight for Cohort II was extracted directly from hospital delivery records. Birthweight was routinely measured within 1 h of delivery using an electronic weight scale (accuracy: 0.01 kg). Newborns with a birthweight below the 10th percentile or above the 90th percentile of gestational age- and sex-specific growth curves [29] were classified as small for gestational age (SGA) or large for gestational age (LGA), respectively. The gestational age at birth was estimated based on the last menstrual period and subsequently confirmed using first-trimester ultrasound. Newborns with a birthweight ≥ 4000 g were classified as macrosomia, and those with a birthweight < 2500 g were classified as LBW.

Covariates for Cohort I were extracted from the RCT database, while those for Cohort II were collected using a structured questionnaire. Both cohorts had available data on maternal sociodemographic and clinical characteristics, including age, ethnicity (Han/other), education level (primary school, lower/secondary school/high school, or higher), occupation (farmer/other), height, early-pregnancy weight, gestational age at blood collection and at delivery, GDM (yes/no), pregnancy anemia (yes/no), and infant sex (male/female). Early-pregnancy BMI was calculated as early-pregnancy weight (kg) divided by height squared (m^2^) and categorized into three groups: underweight (<18.5 kg/m^2^), normal weight (18.5–23.9 kg/m^2^), or overweight/obesity (≥24.0 kg/m^2^). Plasma folate concentrations in blood samples from both cohorts were previously quantified.

### 2.4. Statistical Analysis

Descriptive statistics were used to summarize maternal and perinatal characteristics for each dataset. Spearman correlation was used to calculate the correlation coefficients of choline and betaine concentrations between maternal plasma in late pregnancy and umbilical cord blood. Fractional polynomial regression models were first employed to examine the linear or non-linear relationships between plasma choline and betaine concentrations and birthweight. As the linear relationships were identified, crude and multivariable linear regression analyses were used for subsequent modeling. Crude and multivariable logistic regression models were used to estimate the odds ratios (ORs) and 95% confidence intervals (95% CIs) for binary outcomes (LGA, SGA, macrosomia, or LBW) according to quintiles of choline and betaine concentrations. In multivariable models, adjusted covariates included maternal age, early-pregnancy BMI, education level, occupation, ethnicity, GDM, pregnancy anemia, gestational age at delivery, infant sex, and plasma folate concentration, and choline and betaine concentrations were mutually adjusted. Missing values were imputed using multiple imputation analyses. Given the differences in the two cohorts (such as time periods), cohort was additionally adjusted in multivariable models.

Given that infant birthweight is likely influenced by maternal BMI [30] and GDM status [31], subgroup analyses were performed stratified by maternal BMI (underweight, normal weight, or overweight/obesity) and GDM status (yes or no). The modifying effects of these factors were evaluated by including interaction terms in the multivariable models.

All statistical analyses were performed using R software (version 4.5.1; Copyright © 1998–2020, Kurt Hornik, San Diego, CA, USA). Statistical significance was set as a two-sided *p* < 0.05.

## 3. Results

Maternal and perinatal characteristics are summarized in Table 1. Most mothers had a normal weight in early pregnancy, were of Han ethnicity, did not have GDM or anemia during pregnancy, and gave birth to infants with a normal birthweight (2500–3999 g).

The median (P25, P75) maternal plasma choline and betaine concentrations in late pregnancy were 12.34 (10.13, 14.78) μmol/L and 14.99 (12.01, 18.36) μmol/L, respectively, both lower than those in umbilical cord blood (29.98 [24.74, 35.93] and 31.14 [25.56, 37.28] μmol/L, respectively). Maternal and cord blood choline and betaine concentrations showed no significant changes across gestational age at sampling (Supplementary Figure S1), maternal BMI group, or GDM status (Supplementary Figure S2). The Spearman correlation coefficients between choline and betaine were 0.51 in maternal plasma and 0.38 in cord blood, and those between maternal and umbilical cord levels were 0.11 for choline, 0.19 for betaine, and 0.15 for their sum. The quintile distributions of choline and betaine concentrations are detailed in Supplementary Table S2.

**Figure 2:**
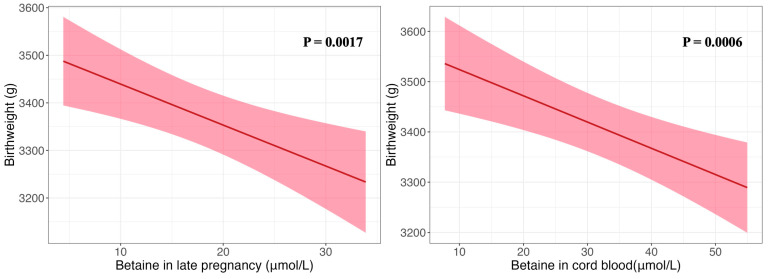
Association of maternal late-pregnancy and cord blood betaine concentration with birthweight.

**Figure 3:**
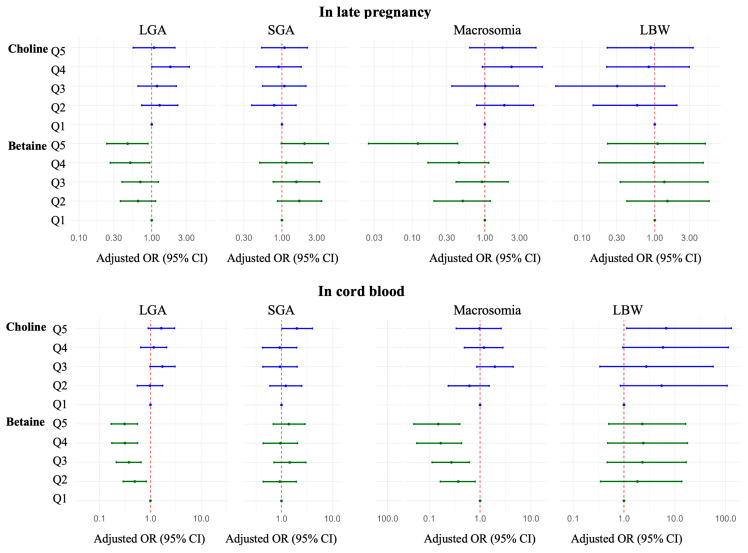
Forest plots of logistic regression of maternal late-pregnancy and cord blood choline and betaine concentration quintiles with binary birthweight outcomes. Multivariable-adjusted ORs (indicated by the dots) and 95% CIs (indicated by horizontal lines). Abbreviations: CI, confidence interval; LBW, low birthweight; LGA, large for gestational age; OR, odds ratio; and SGA, small for gestational age.

The relationships between maternal and cord blood choline and betaine concentrations and birthweight are shown in Table 2. In multivariable linear regression models, each 1 μmol/L increment in late-pregnancy or cord blood betaine concentration was associated with 9.87 g (95% CI: −16.08, −3.66 g) or 5.29 g (95% CI: −8.52, −2.06 g) lower birthweight, respectively (Figure 2). In multivariable logistic regression models using quintile-based exposure grouping, compared with the lowest quintile, the highest quintiles of late-pregnancy and cord blood betaine concentrations were associated with lower risks of LGA (adjusted ORs = 0.47 [95% CI: 0.24, 0.90] and 0.31 [95% CI: 0.17, 0.56]) and macrosomia (adjusted ORs = 0.12 [95% CI: 0.03, 0.43] and 0.15 [95% CI: 0.05, 0.40]). The risks of SGA and LBW in the highest quintile groups were not increased (Figure 3 and Supplementary Table S3). No significant associations were observed between choline concentrations at any stage and birthweight outcomes (late pregnancy: 2.08 g [95% CI: −5.69, 9.84 g] and cord blood: −0.92 g [95% CI: −4.33, 2.48 g]). The results were the same in sensitivity analysis when excluding preterm infants (Supplementary Table S4 and Supplementary Figure S3).

In subgroup analyses stratified by maternal early-pregnancy BMI and GDM status, the associations persisted across most subgroups, with the associations involving cord blood betaine being particularly robust. Additionally, the magnitude of these associations appeared stronger in subgroups of maternal overweight/obesity or GDM, albeit the interaction effects did not reach statistical significance (Supplementary Table S5). For instance, the relationship between cord blood betaine concentration and birthweight was more pronounced in pregnancies with overweight/obese mothers (β = −7.91 g per 1 μmol/L increment, 95% CI: −14.00, −1.82 g) than in those with underweight or normal-weight mothers (P for interaction = 0.207), and it was more pronounced in pregnancies with GDM (β = −12.57 g, 95% CI: -21.47, −3.67 g) than in those without (*P* for interaction = 0.240). Similar results were observed when quintiles of exposures and binary outcomes were employed (Supplementary Table S6). Notably, these results merit further investigation, as the interaction terms lacked statistical significance.

## 4. Discussion

To our knowledge, this is the first study to explore the associations of maternal plasma choline and betaine concentrations in late pregnancy, as well as cord blood concentrations, with birthweight in a Chinese population. We observed strong inverse associations between late-pregnancy and cord blood betaine concentrations and birthweight. Compared with the lowest quintile, the highest quintiles of late-pregnancy and cord blood betaine concentrations were associated with 53% and 69% lower risks of LGA and 88% and 85% lower risks of macrosomia, respectively. These associations, particularly those involving cord blood betaine, remained robust in pregnancies with maternal overweight/obesity or GDM. No significant associations were observed for choline at any stage.

Choline and betaine concentrations in late pregnancy and cord blood varied markedly across study populations. For instance, late-pregnancy choline and betaine concentrations were 8.0 μmol/L and 10.0 μmol/L in Americans [32], 12.3 μmol/L and 9.5 μmol/L in the Irish [33], and 15.3 μmol/L and 14.1 μmol/L in Canadians [34], which was similar to our study population (12.34 μmol/L and 14.99 μmol/L, respectively). These differences could be attributed to maternal ethnicity, dietary patterns, assay methods, and the specific gestational weeks at blood sampling. However, a consistent finding across studies was that cord blood concentrations of these nutrients were higher than those in late pregnancy. For example, cord blood choline and betaine concentrations were 35.0 μmol/L and 30.0 μmol/L in Americans [32], 36.6 μmol/L and 21.1 μmol/L in the Irish [33], and 36.4 μmol/L and 29.4 μmol/L in Canadians [34]. Similarly, the corresponding concentrations in our study were 29.98 μmol/L and 31.14 μmol/L.

We did not observe significant associations of maternal and cord blood choline concentrations with birthweight in multivariable models, similar to previous studies [22,25]. However, one small study reported contrasting findings: among infants of GDM mothers, cord blood choline concentration was inversely correlated with birthweight (r = −0.363, *P* = 0.017) (*n* = 54) [26]. Notably, in our study, crude analysis revealed a significant inverse association between cord blood choline and birthweight, which turned insignificant after adjusting for multiple covariates, including betaine, indicating that betaine may act as a potential mediator in the choline–birthweight relationship.

We observed strong inverse associations between late-pregnancy and cord blood betaine concentrations and birthweight. Previous studies have reported inconsistent results on the association between maternal late-pregnancy betaine concentration and birthweight. Two studies from the Netherlands [25,35] did not find any significant associations, while two other studies identified inverse associations. Specifically, the Singapore GUSTO cohort study [23] reported that each 5 μmol/L increase in maternal betaine concentration at 26–28 weeks of gestation was associated with 57.6 g lower birthweight. A small Chinese study (*n* = 115) [22] also showed that higher maternal betaine concentrations were associated with lower birthweight (β = −130.3 g per SD increment). Consistent with these two studies, our results demonstrated that each 1 μmol/L increase in late-pregnancy betaine concentration was associated with 11.13 g lower birthweight. Additionally, we found that each 1 μmol/L increase of cord blood betaine concentration was associated with 5.86 g lower birthweight. This finding aligns with a prior Dutch study, which showed that higher cord blood betaine concentration was associated with lower birthweight (β = −65 g per SD increment) [25], and a small Mexican study (*n* = 54), which showed that cord blood betaine concentration was inversely correlated with birthweight in infants born to GDM women (r = −0.425, *P* = 0.007) [26].

Notably, our study extends the previous findings by demonstrating that higher betaine concentrations in late pregnancy and cord blood confer a protective effect against macrosomia. As compared with the lowest quintile, the highest quintiles of late-pregnancy and cord blood betaine concentrations were associated with 53% and 69% lower risks of LGA, as well as 88% and 85% lower risks of macrosomia, respectively. These findings highlight important clinical and public health implications, particularly for pregnancies at high risk of macrosomia, such as those complicated by maternal overweight/obesity or GDM.

Our subgroup analyses showed that the inverse association between betaine concentration and birthweight remained robust in high-risk pregnancies, specifically for cord blood betaine concentration rather than late-pregnancy betaine concentration. This is likely because cord blood betaine tended to reflect placental transfer efficiency and fetal metabolic utilization [25], thereby more accurately representing the actual fetal betaine exposure. Although associations appeared stronger in overweight/obese and GDM subgroups, these findings are exploratory given that the interaction term did not reach statistical significance; thus, they warrant further investigation.

The inverse association between betaine and fetal overgrowth may be mediated via three key mechanisms. First, higher betaine could downregulate the expression of placental nutrient transporters (e.g., SLC44A1, GLUT1, and FATP4) [13,15,18,36], thereby preventing excessive maternal–fetal macronutrient transfer, enhancing placental glycogen and lipid storage, avoiding excessive nutrient accumulation in the fetus, and curbing fetal overgrowth. Second, as a methyl donor, betaine participates in DNA methylation and regulates fatty acid metabolism by upregulating genes related to fatty acid oxidation (e.g., PPAR α) and downregulating those that promote lipogenesis (e.g., FASN) [16,17,37,38]. Third, betaine can reduce the incidence of fetal hyperinsulinemia, alleviate fetal insulin and leptin resistance, and enhance fetal lipid metabolism [19,20,39]. These pathways inhibit excessive accumulation of fetal fat and decrease the risk of macrosomia.

The primary strength of our study was that the nutrient concentrations were measured at two key time points (late pregnancy and cord blood at delivery), enabling a relatively comprehensive assessment of exposure during critical windows of fetal development. Other strengths included a relatively large sample size, adjustment for major confounders, prospective study design, and use of biomarkers as exposure variables; this approach avoided recall bias inherent to dietary questionnaires and more accurately reflected nutrient bioavailability and metabolic processes. Nonetheless, several limitations should be acknowledged. First, samples from the first and second trimester were not collected and analyzed, which precluded the tracking of dynamic changes in choline and betaine concentrations across pregnancy and exploration of their cumulative effects on fetal growth. Second, temporal and methodological confounding could not be entirely ruled out given that the two cohorts were established at distinct periods (Cohort I: 2006–2009; Cohort II: 2023), even after adjustments were made in multivariable models. Third, we did not explore the potential mechanisms involved in homocysteine degradation and S-adenosylmethionine (SAM) formation via betaine homocysteine methyltransferase reactions. Fourth, the absence of long-term follow-up data prevented evaluation of whether the betaine–birthweight association extended to childhood obesity, type 2 diabetes, or other metabolic outcomes in later life. Finally, the study sample was restricted to Chinese participants from Hebei and Shandong provinces, which could limit the generalizability of our findings to other ethnic groups or geographical regions.

## 5. Conclusions

In conclusion, this study demonstrated that higher maternal late-pregnancy and cord blood betaine concentrations were associated with lower birthweight and reduced risk of macrosomia in Chinese populations. These associations persisted in pregnancies with higher risk of macrosomia, such as those with maternal overweight/obesity or GDM. These findings support a regulatory role of betaine in fetal growth and highlight its potential as an intervention target for preventing fetal overgrowth in high-risk pregnancies. Future prospective cohort studies that follow women from early pregnancy through delivery are warranted to elucidate how maternal betaine concentrations—both throughout gestation and in cord blood—influence fetal growth trajectories.

## Figures and Tables

**Table 1:** Maternal and perinatal characteristics and plasma concentrations of nutrients.

	Mother–Infant Dyads (*n* = 988)
Maternal age (year)	25.65 ± 4.59
Early-pregnancy BMI (kg/m^2^)	
<18.5 (underweight)	68 (6.88)
18.5 to 24 (normal weight)	630 (63.77)
≥24 (overweight/obesity)	290 (29.35)
Education	
Primary school or lower	311 (31.48)
Secondary school	571 (57.79)
High school or higher	102 (10.32)
Missing	4 (0.40)
Occupation	
Farmer	614 (62.15)
Other	369 (37.35)
Missing	5 (0.51)
Ethnicity	
Han	976 (98.78)
Other	7 (0.71)
Missing	5 (0.51)
GDM	
No	867 (87.75)
Yes	121 (12.25)
Anemia	
No	754 (76.31)
Yes	170 (17.21)
Missing	64 (6.48)
Birthweight (g)	3305 (3050, 3550; min: 1780; max: 5200)
<2500 (LBW)	23 (2.33)
2500 to 4000 (normal weight)	910 (92.10)
≥4000 (macrosomia)	55 (5.57)
Gestational Age (weeks)	39.00 (38.04, 40.00; min: 32; max: 47)
Sex	
Male	487 (49.29)
Female	501 (50.71)
SGA	
No	890 (90.08)
Yes	98 (9.92)
LGA	
No	829 (83.91)
Yes	159 (16.09)
Plasma nutrients concentrations (μmol/L) in late pregnancy	
Choline	12.34 (10.13, 14.78)
Betaine	14.99 (12.01, 18.36)
Folate	10.65 (7.37, 17.69)
Plasma nutrients concentrations (μmol/L) in cord blood	
Choline	29.98 (24.74, 35.93)
Betaine	31.14 (25.56, 37.28)
Folate	27.73 (19.87, 38.48)

Statistics are displayed as mean ± SD for maternal age, median (P25, P75) for other continuous variables, and n (%) for categorical variables. Abbreviations: BMI, body mass index; GDM, gestational diabetes mellitus; LGA, large for gestational age; SD, standard deviations; and SGA, small for gestational age.

**Table 2:** Associations of maternal and cord blood choline and betaine concentrations (µmol/L) with birthweight (g).

	Crude β (95% CI)	*P*	Adjusted β (95% CI) ^1^	*P*
Late pregnancy				
Choline	0.25 (−6.61, 7.11)	0.944	2.08 (−5.69, 9.84)	0.6002
Betaine	−6.19 (−11.65, −0.74)	0.0261	−9.87 (−16.08, −3.66)	0.0019
Cord blood				
Choline	−4.82 (−8.12, −1.52)	0.0042	−0.92 (−4.33, 2.48)	0.5948
Betaine	−6.18 (−9.26, −3.10)	<0.0001	−5.29 (−8.52, −2.06)	0.0013

^1^: Adjusted for cohort, maternal age, pre-pregnancy BMI, education level (primary school or lower/secondary school/high school and above), occupation (famer/other), ethnicity (Han/other), gestation, infant sex, pregnancy diabetes (yes/no), pregnancy anemia (yes/no), and plasma folate concentration; choline and betaine were mutually adjusted. Abbreviation: CI, confidence interval.

## Data Availability

Data described in the manuscript may be made available upon request pending necessary author and ethical approvals due to privacy.

## Supplementary Materials

The following supporting information can be downloaded at https://www.mdpi.com/article/10.3390/nu18091456/s1. Table S1: Characteristics of included and original participants; Table S2: Quintiles of choline and betaine plasma concentrations; Table S3: Logistic regression of quintiles of choline and betaine concentrations with binary birthweight outcomes; Table S4: Associations of maternal and cord blood choline and betaine concentrations (µmol/L) with birthweight (g) after excluding premature infants. Table S5: Associations of late-pregnancy and cord blood betaine concentrations (µmol/L) with birthweight in subgroup analysis; and Table S6: Associations of quintiles of late-pregnancy and cord blood betaine concentrations (µmol/L) with binary birthweight outcomes in subgroup analysis. Figure S1: Scatter plots of choline/betaine in late pregnancy and cord blood across gestational weeks. Figure S2: Box plots of choline/betaine in late pregnancy and cord blood across strata of BMI/GDM. Figure S3: Forest plots of logistic regression of maternal late-pregnancy and cord blood choline and betaine concentrations quintiles with binary birthweight outcomes after excluding premature infants.

## Abbreviations

The following abbreviations are used in this manuscript:

BMI	body mass index
CI	confidence interval
GDM	gestational diabetes mellitus
IQR	interquartile range
LBW	low birthweight
LGA	large for gestational age
OR	odds ratio
RCT	randomized controlled trial
SD	standard deviations
SGA	small for gestational age
UPLC/MS–MS	ultra-performance liquid chromatography–mass spectrometry
