# Supplementary material for: Association of Maternal and Cord Blood Choline and Betaine Concentrations with Birthweight: A Prospective Mother–Infant Cohort Study
Source: Nutrients. 2026 May 1;18(9):1456. doi: 10.3390/nu18091456 (PMC13164811; doi:10.3390/nu18091456)
Supplement: Supplementary file 1 [file nutrients-18-01456-s001.zip › nutrients-4261607-supplementary.pdf]

Supplementary Table S1. Characteristics of included and original participants.

**Characteristics**	Overall (n = 17613)	Included (n = 690)
Maternal Age (year)	23.62 (2.83)	23.86 (3.32)
Education		
Primary school or lower	3204 (18.2)	55 (8.0)
Secondary school	14130 (80.2)	534 (77.4)
High school or higher	279 (11.6)	101 (14.6)
Occupation		
Famer	16018 (90.9)	612 (88.7)
Other	1595 (9.1)	78 (11.3)
Early-pregnancy BMI (Kg/m^2)		
<18.5 (underweight)	1568 (8.9)	41 (5.9)
18.5 to 24 (normal weight)	12996 (73.8)	475 (68.8)
>=24 (overweight/obesity)	3049 (17.3)	174 (25.2)
Gestational Age (weeks)	39.6 (1.67)	39.31(1.64)
Birthweight (g)	3300 (386)	3296 (422)
Sex		
Male	9240 (52.5)	317 (45.9)
Female	8365 (47.5)	373 (54.1)
Missing	8 (0.0)	-

Statistics display as mean ± SD for continuous variables, and n (%) for categorical variables.

Abbreviation: BMI, body mass index; SD, standard deviations.

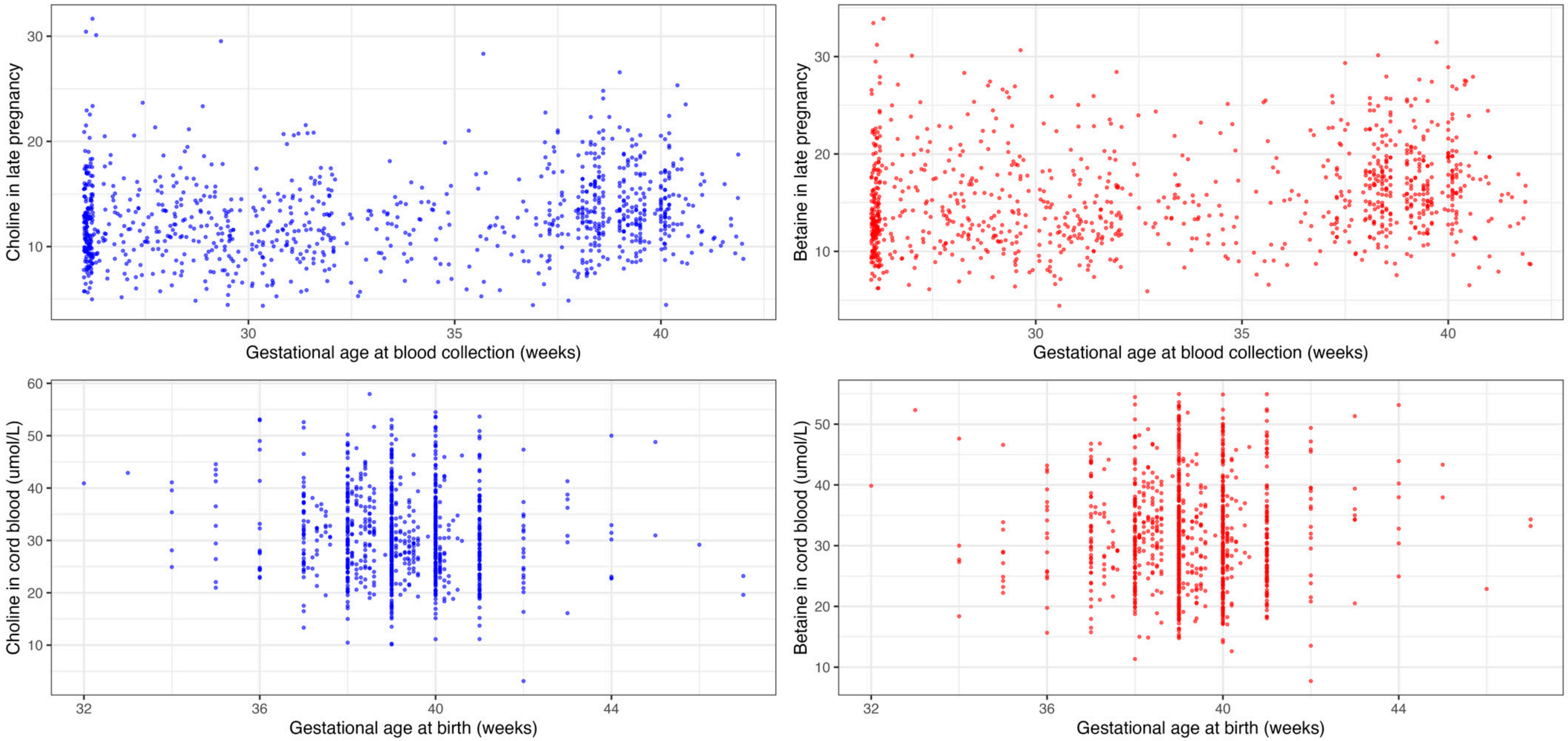

Supplementary Figure S1: Scatter plots of choline/betaine in late pregnancy and cord blood across gestational weeks

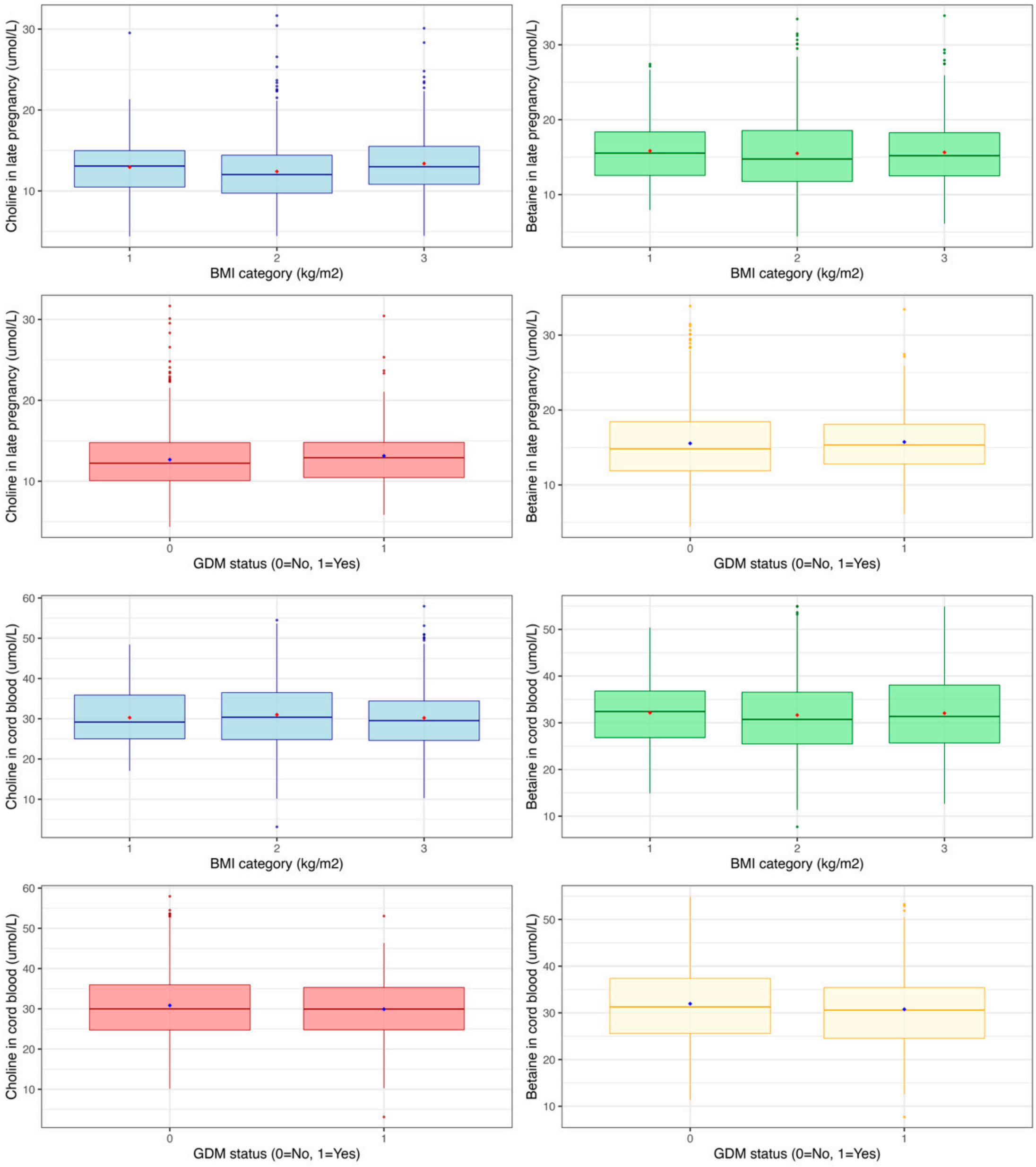

Supplementary Figure S2: Box plots of choline/betaine in late pregnancy and cord blood across strata of BMI/GDM

Supplementary Table S2. Quintiles of choline and betaine plasma concentrations.

	Q1	Q2	Q3	Q4	Q5
Late pregnancy					
Choline (μmol/L)	4.38-9.60	9.62-11.70	11.71-13.47	13.48-16.63	16.67-55.29
Betaine (μmol/L)	4.42-11.67	11.70-13.92	13.93-16.55	16.57-19.78	19.79-33.93
Umbilical cord blood					
Choline (μmol/L)	3.14-23.98	23.99-27.77	27.78-31.84	31.85-37.47	37.49-41.23
Betaine (μmol/L)	7.71-24.28	24.29-28.97	28.98 -33.27	33.27-39.01	39.05-54.96

Supplementary Table S3. Logistic regression of quintiles of choline and betaine concentrations with binary birthweight outcomes[1].

	LGA		SGA		Macrosomia		LBW	
	Adjusted OR (95% CI)	P	Adjusted OR (95% CI)	P	Adjusted OR (95% CI)	P	Adjusted OR (95% CI)	P
Late pregnancy								
Choline Q1	Ref.		Ref.		Ref.		Ref.	
Choline Q2	1.29 (0.73, 2.29)	0.3849	0.78 (0.38, 1.55)	0.4791	1.86 (0.78, 4.70)	0.1716	0.58 (0.14, 2.02)	0.4020
Choline Q3	1.19 (0.65, 2.19)	0.5748	1.08 (0.54, 2.14)	0.8288	1.02 (0.35, 2.91)	0.9669	0.30 (0.04, 1.36)	0.1533
Choline Q4	1.81 (1.01, 3.30)	0.0507	0.90 (0.44, 1.83)	0.7699	2.32 (0.92, 6.18)	0.0796	0.82 (0.21, 2.96)	0.7584
Choline Q5	1.08 (0.56, 2.10)	0.8118	1.10 (0.53, 2.26)	0.8028	1.74 (0.61, 5.02)	0.2970	0.84 (0.21, 3.23)	0.7998
Betaine Q1	Ref.		Ref.		Ref.		Ref.	
Betaine Q2	0.65 (0.37, 1.13)	0.1274	1.73 (0.87, 3.51)	0.1195	0.49 (0.20, 1.17)	0.1119	1.50 (0.41, 5.63)	0.5373
Betaine Q3	0.70 (0.39, 1.24)	0.2257	1.56 (0.75, 3.28)	0.2316	0.92 (0.41, 2.11)	0.8554	1.37 (0.34, 5.48)	0.6480
Betaine Q4	**0.51 (0.27, 0.94)**	**0.0335**	1.14 (0.49, 2.59)	0.7571	0.44 (0.16, 1.13)	0.0910	0.99 (0.17, 4.77)	0.9906
Betaine Q5	**0.47 (0.24, 0.90)**	**0.0239**	2.02 (0.96, 4.29)	0.0647	**0.12 (0.03, 0.43)**	**0.0027**	1.14 (0.23, 5.19)	0.8639
Umbilical cord blood								
Choline Q1	Ref.		Ref.		Ref.		Ref.	
Choline Q2	0.98 (0.55, 1.74)	0.9422	1.12 (0.54, 2.34)	0.7522	0.62 (0.24, 1.52)	0.3052	5.56 (0.85, 108.73)	0.1241
Choline Q3	1.73 (0.98, 3.06)	0.0588	0.93 (0.43, 2.02)	0.8604	1.96 (0.87, 4.52)	0.1071	2.78 (0.34, 57.78)	0.3862
Choline Q4	1.17 (0.65, 2.10)	0.0606	1.07 (0.51, 2.29)	0.8498	1.19 (0.49, 2.82)	0.6972	5.81 (0.94, 112.34)	0.1104
Choline Q5	1.68 (0.92, 3.07)	0.0925	1.90 (0.97, 3.88)	0.0678	1.02 (0.35, 2.75)	0.9687	6.96 (1.00, 133.98)	0.0767
Betaine Q1	Ref.		Ref.		Ref.		Ref.	
Betaine Q2	**0.49 (0.29, 0.83)**	**0.0082**	0.93 (0.44, 1.97)	0.8535	**0.38 (0.17, 0.80)**	**0.0142**	1.79 (0.33, 13.34)	0.5113
Betaine Q3	**0.37 (0.21, 0.65)**	**0.0006**	1.42 (0.69, 2.94)	0.3413	**0.28 (0.11, 0.62)**	**0.0027**	2.26 (0.46, 16.42)	0.3470
Betaine Q4	**0.31 (0.17, 0.56)**	**0.0001**	0.92 (0.43, 2.01)	0.8394	**0.17 (0.06, 0.44)**	**0.0005**	2.35 (0.46, 17.36)	0.3312

Betaine Q5	**0.31 (0.17, 0.56)**	**0.0001**	1.39 (0.68, 2.89)	0.3693	**0.15 (0.05, 0.40)**	**0.0003**	2.23 (0.49, 15.88)	0.3444

[1]: Adjusted for cohort, maternal age, pre-pregnancy BMI, education level (primary school or lower/secondary school/high school and above), occupation (famer/other), ethnic (Han/other), gestation, infant sex, pregnancy diabetes (yes/no), pregnancy anemia (yes/no), plasma folate concentration and mutually adjusted for choline and betaine.

Abbreviation: CI, confidence interval; LBW, low birthweight; LGA, large-for-gestational-age; OR, odds ratio; SGA, small-for-gestational-age.

Supplementary Table S4. Associations of maternal and cord blood choline and betaine concentrations (μmol/l) with birthweight (g) after excluding premature infants.

	Crude β (95% CI)	P	Adjusted β (95% CI) [1]	P
Late pregnancy				
Choline (μmol/L)	0.18 (-6.67, 7.03)	0.959	4.52 (-3.30, 12.34)	0.2570
Betaine (μmol/L)	**-5.74 (-11.23, -0.26)**	**0.0261**	**-10.98 (-17.33, -4.63)**	**0.0007**
Umbilical cord blood				
Choline (μmol/L)	**-4.45 (-7.78, -1.12)**	**0.0088**	-1.46 (-4.95, 2.04)	0.4132
Betaine (μmol/L)	**-6.10 (-9.17, -3.01)**	**<0.0001**	**-5.81 (-9.07, -2.55)**	**0.0005**

[1]: Adjusted for cohort, maternal age, pre-pregnancy BMI, education level (primary school or low-er/secondary school/high school and above), occupation (famer/other), ethnic (Han/other), gesta-tion, infant sex, pregnancy diabetes (yes/no), pregnancy anemia (yes/no), plasma folate concentra-tion; and choline and betaine were mutually adjusted.

Abbreviation: CI, confidence interval.

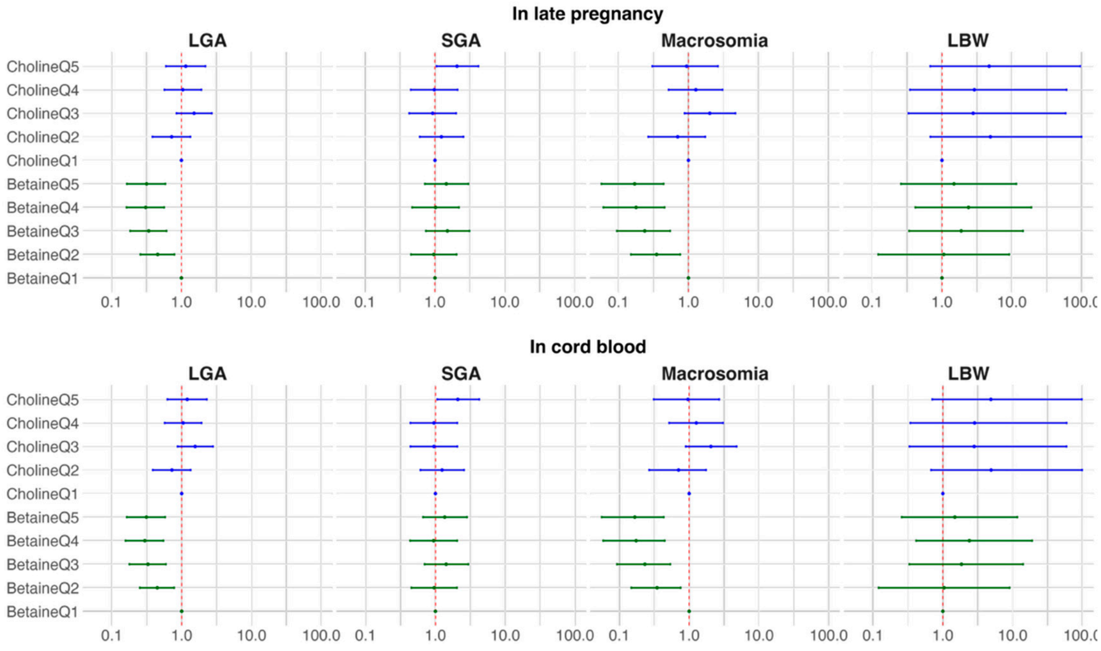

Supplementary Figure S3. Forest plots of logistic regression of maternal late-pregnancy and cord blood choline and betaine concentrations quintiles with binary birthweight outcomes after excluding premature infants. Multivariable-adjusted ORs (indicated by the dots) and 95% CIs (indicated by horizontal lines). Abbreviation: CI, confidence interval; LBW, low birthweight; LGA, large-for-gestational-age; OR, odds ratio; SGA, small-for-gestational-age.

Supplementary Table S5. Associations of late-pregnancy and cord blood betaine concentrations (μmol/l) with birthweight in subgroup analysis

	Betaine (μmol/L) in late pregnancy		Betaine (μmol/L) in cord blood	
	Adjusted β (95% CI)	P for interaction	Adjusted β (95% CI)	P for interaction
**Early-pregnancy BMI（Kg/m²）**[1]				
Underweight	-22.26 (-46.76, 2.24)	0.1635	4.10 (-9.97, 18.17)	0.2069
Normal weight	**-14.38 (-21.77, -6.99)**		**-5.68 (-9.69, -1.68)**	
Overweight/obesity	-1.03 (-14.10, 12.04)		**-7.91 (-14.00, -1.82)**	
**GDM**[2]				
No	**-10.15 (-16.57, -3.73)**	0.6607	**-5.16 (-8.60, -1.71)**	0.2395
Yes	-18.50 (-41.42, 4.40)		**-12.57 (-21.47, -3.67)**	

[1]: Adjusted for cohort, maternal age, education level (primary school or lower/secondary school/high school and above), occupation (famer/other), ethnic (Han/other), gestation, infant sex, pregnancy diabetes (yes/no) and pregnancy anemia (yes/no), plasma folate concentration and mutually adjusted for choline and betaine.

[2]: Adjusted for maternal age, pre-pregnancy BMI, education level (primary school or lower/secondary school/high school and above), occupation (famer/other), ethnic (Han/other), gestation, infant sex, and pregnancy anemia (yes/no), plasma folate concentration and mutually adjusted for choline and betaine.

Abbreviation: BMI, body mass index; CI, confidence interval; GDM, gestational diabetes mellitus.

Supplementary Table S6. Associations of quintiles of late-pregnancy and cord blood betaine concentrations (μmol/l) with binary birthweight outcomes in subgroup analysis

	LGA			Macrosomia		
	Q1	Q5 Adjusted OR (95% CI)	P for interaction	Q1	Q5 Adjusted OR (95% CI)	P for interaction
**Betaine (μmol/L) in Late pregnancy**						
**Early-pregnancy BMI（Kg/m²）**[1]						
Underweight	Ref.	-		Ref.	-	
Normal weight	Ref.	**0.37 (0.16, 0.82)**	0.2795	Ref.	**-**	0.8600
Overweight/obesity	Ref.	0.75 (0.21, 2.71)		Ref.	**0.12 (0.01, 0.91)**	
**GDM**[2]						
No	Ref.	**0.45 (0.23, 0.89)**	0.9968	Ref.	**0.17 (0.03, 0.60)**	0.9843
Yes	Ref.	0.49 (0.06, 3.98)		Ref.	-	
**Betaine (μmol/L) in cord blood**						
**Early-pregnancy BMI（Kg/m²）**[1]						
Underweight	Ref.	-		Ref.	-	
Normal weight	Ref.	**0.30 (0.14, 0.61)**	0.5545	Ref.	**0.16 (0.05, 0.49)**	0.2054
Overweight/obesity	Ref.	**0.32 (0.10, 0.96)**		Ref.	**0.09 (0.003, 0.65)**	
**GDM**[2]						
No	Ref.	**0.25 (0.12, 0.48)**	0.4439	Ref.	**0.17 (0.05, 0.50)**	0.9855
Yes	Ref.	**0.16 (0.02, 0.86)**		Ref.	-	

[1]: Adjusted for cohort, maternal age, education level (primary school or lower/secondary school/high school and above), occupation (famer/other), ethnic (Han/other), gestation, infant sex, pregnancy diabetes (yes/no) and pregnancy anemia (yes/no), plasma folate concentration and mutually

adjusted for choline and betaine.

[2]: Adjusted for maternal age, pre-pregnancy BMI, education level (primary school or lower/secondary school/high school and above), occupation (famer/other), ethnic (Han/other), gestation, infant sex, and pregnancy anemia (yes/no), plasma folate concentration and mutually adjusted for choline and betaine.

Abbreviation: BMI, body mass index; CI, confidence interval; GDM, gestational diabetes mellitus; LGA, large-for-gestational-age; OR, odds ratio.
